# Characterization of the complete chloroplast genome of *Jatropha curcas* var. *nigroviensrugosus*

**DOI:** 10.1080/23802359.2021.1993101

**Published:** 2022-01-24

**Authors:** Lu Chun-Yun, Zhao Yang, Wang Xiu-rong

**Affiliations:** a College of Forestry, Guizhou University, Guiyang, Guizhou, China; b Institute for Forest Resources & Environment of Guizhou, Guizhou University, Guiyang, Guizhou, China; c Key Laboratory of Forest Cultivation in Plateau Mountain of Guizhou Province, Guiyang, Guizhou, China; d Key Laboratory of Plant Resource Conservation and Germplasm Innovation in Mountainous Region (Ministry of Education), Guizhou University, Guiyang, Guizhou, China

**Keywords:** *Jatropha nigroviensrugosus*, complete chloroplast genome, phylogenetic analysis

## Abstract

As a new variety of *Jatropha curcas* L., *Jatropha curcas* var. *nigroviensrugosus* has high development and utilization values because of its high flowering and fruiting rates and yield. In this study, the complete chloroplast (cp) genome of *J. nigroviensrugosus* was assembled using Illumina sequencing data. Results revealed that its cp genome is 170,811 bp in length and has 106 unique genes, including 76 protein-coding genes, 26 tRNA genes and 4 rRNA genes. Phylogenetic analysis indicated that *J. nigroviensrugosus* was closely related to *J. curcas*.

*Jatropha curcas* var. *nigroviensrugosus* CV Yang, which belongs to Euphorbiaceae, is a discovered variant of *J. curcas* and developed via selective cross-breeding; its main features are downward cotyledons and true leaves and bulged leaf tissues or wrinkled leaves (Yang 2013; Chengyuan 2013; Chengyuan et al. 2015). As an energy-producing tree species, it has high development and utilization values because of its high flowering and fruiting rates and yield.

Chloroplast (cp) genome has a conserved structure and is often used to identify the phylogenetic relationship of species, the genetic relationship between species and the homologous relationship of molecular traits. In this study, total genomic DNA was extracted from fresh leaves of a seedling of *J. nigroviensrugosus* was collected from Zhe Gan, A Long City, Guizhou Province, China (E: 105.4535971005249 N: 24.918747530829368) (Collector was *Doc.* Feng Xiao, Email: maplexiao594@gmail.com), total genomic DNA was extracted by using an EasyPure^®^ plant genomic DNA kit (TransGen Biotech, Beijing, China). A short-insert library (insert size of 400 bp) was prepared and sequenced with the Illumina NovaSeq platform. The seed specimen was placed at the Institute for Forest Resources and Environment of Guizhou (accessions *No.* JN-001-1). The genome sequence was assembled with GetOrganelle (Jin et al. 2020), and the cp was annotated in CPGAVAS2 (Shi et al. 2019). The annotated cp genome sequence and raw reads were deposited into Genbank with the accession numbers MW023058 and PRJNA737608, respectively.

The size of the cp genome of *J. nigroviensrugosus* was 170,811 bp, including a large single-copy (LSC) region of 98,485 bp, a small single-copy (SSC) region of 17,888 bp and two reverse repeated regions (IRa and IRb) of 27,219 bp in length. The GC content of the cp genome was 35.42%. The cp genome of *J. nigroviensrugosus* contained 106 distinct genes, including 76 protein-coding genes, 26 tRNA genes and 4 rRNA genes. Twenty plant species were used in phylogenetic analysis to explore the phylogenetic position of *J. nigroviensrugosus*. MAFFT (Katoh and Standley 2017) was utilized to align the sequences, and a maximum likelihood tree was constructed with iqtree (Figure 1, Nguyen et al. 2015). Phylogenetic analysis showed that *J. nigroviensrugosus* was closely related to *J. curcas*.

**Figure 1.**
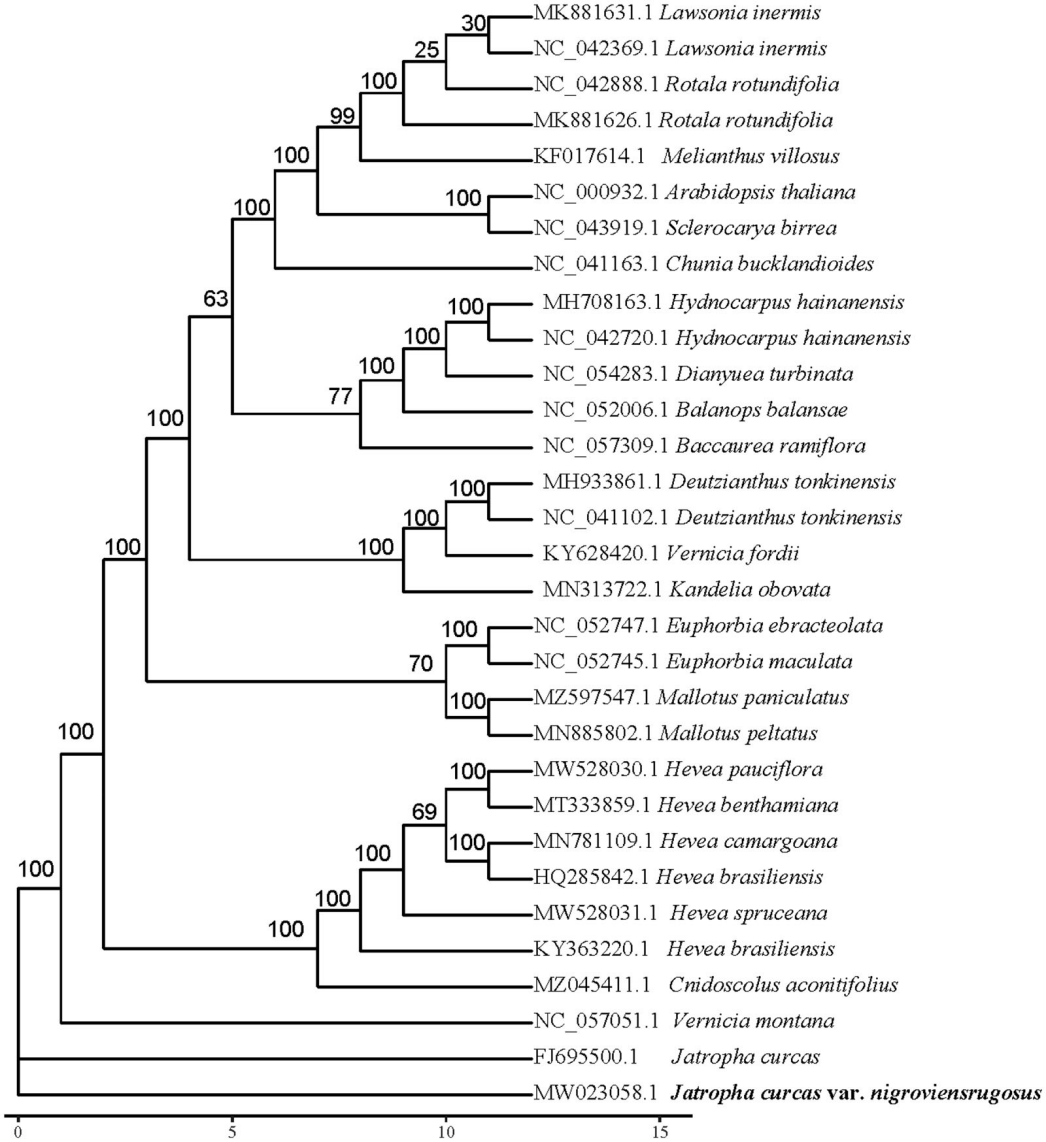
Phylogenetic tree based on 31 complete chloroplast genome sequences.

## Data Availability

The genome sequence data supporting the findings of this study are openly available in GenBank of NCBI (https://www.ncbi.nlm.nih.gov/) under the Accession No. MW023058.1. The associated BioProject, SRA and BioSample numbers are PRJNA737608, SRR14816991 and SAMN19700807, respectively.
